# Metal-Semiconductor-Metal Near-Ultraviolet (~380 nm) Photodetectors by Selective Area Growth of ZnO Nanorods and SiO_2_ Passivation

**DOI:** 10.1186/s11671-016-1541-3

**Published:** 2016-07-15

**Authors:** Soo Hyun Lee, Sang Hun Kim, Jae Su Yu

**Affiliations:** Department of Electronics and Radio Engineering, Kyung Hee University, 1732 Deogyeong-daero, Giheung-gu, Yongin-si, Gyeonggi-do 446-701 Republic of Korea

**Keywords:** Zinc oxide nanorods, Photodetectors, Near-ultraviolet, Selective area growth, Passivation

## Abstract

Metal-semiconductor-metal near-ultraviolet (NUV) photodetectors (PDs) based on zinc oxide (ZnO) nanorods (NRs), operating at *λ* ~ 380 nm, were fabricated using conventional photolithography and hydrothermal synthesis processes. The vertically aligned ZnO NRs were selectively grown in the channel area of PDs. The performance of ZnO NR-based NUV PDs was optimized by varying the solution concentration and active channel width (*W*_ch_). For the fabricated samples, their electrical and photoresponse properties were investigated under the dark state and the illumination at wavelength of ~380 nm, respectively. For the device (*W*_ch_ = 30 μm) with ZnO NRs at 25 mM, the highest photocurrent of 0.63 mA was obtained with the on/off ratio of 1720 at the bias of 5 V. The silicon dioxide passivation was also carried out to improve the photoresponse properties of PDs. The passivated devices exhibited faster rise and reset times rather than those of the unpassivated devices.

## Background

Zinc oxide (ZnO) nanostructures (NSs, i.e., thin films, nanowires, and nanorods) have been extensively investigated in various applications including optoelectronics, transparent electrodes, and sensors due to their high transparency in the visible range, low cost, radiation hardness, and thermal/chemical stabilities [1–12]. Particularly, ZnO is a promising metal oxide semiconductor material for short wavelength (ultraviolet (UV) and blue) applications including photodetectors (PDs), lasers, and light-emitting diodes due to its excellent optical properties such as wide direct bandgap of 3.37 eV and large exciton binding energy of 60 meV at room temperature. In addition, the ZnO NSs have larger surface-to-volume ratio and higher quantum efficiency compared to the bulk ZnO, which makes themselves as a promising candidate for UV PDs [1, 2, 5–9]. However, there is very little information on ZnO homojunction-based UV PDs because undoped ZnO generally exhibits a nature of *n*-type conductivity while *i*- or *p*-type ZnO with high quality and reproducibility is difficult to be obtained [1, 3]. Meanwhile, in most reports on ZnO-based UV PDs, metal-semiconductor-metal (MSM) structures which provide an easy control, stable operation, simple fabrication process, and compatibility with various materials have been employed [1–6].

However, there are still practical limitations to grow ZnO NSs on selected areas of MSM structures. In order to define the selective areas, a conventional photolithography process is required, causing complex fabrication processes with potential damages. Although there has been much effort to grow ZnO NSs efficiently by various methods, these issues still need to be discussed. Meanwhile, hydrothermal synthesis has been considered as a promising method due to its relatively simple process, low working temperature, and short growth time [2, 13–15]. In this work, we investigated the synthesis and characteristics of one-dimensional ZnO nanorods (NRs) hydrothermally formed on the Al-doped ZnO (AZO) layer in MSM structures by the selective area growth and their applications for near-ultraviolet (NUV) PDs. To understand the passivation effect on the photoresponse property, comparative studies between the devices with/without silicon dioxide (SiO_2_) passivation were also carried out.

## Methods

**Fig. 1:**
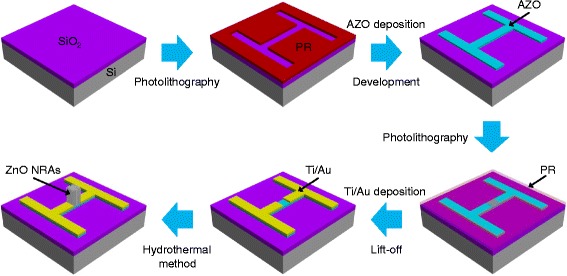
Schematic illustration. Fabrication steps of the NUV PDs in the type of MSM structure by selective area growth of ZnO NRs using conventional photolithography and hydrothermal method

Figure 1 shows the schematic illustration for the fabrication steps of the NUV PDs in the type of MSM structure by selective area growth of ZnO NRs using conventional photolithography and hydrothermal method. The SiO_2_-coated Si substrates were prepared and cleaned in acetone, methanol, and de-ionized (DI) water for 10 min, respectively. The active channel areas were defined by a conventional photolithography process. The 50-nm-thick AZO layer (98 wt% ZnO/2 wt% Al_2_O_3_) was deposited by using a radio-frequency (RF) magnetron sputtering system (KVS-3004, Korea Vacuum Tech., LTD), and then the development process was followed. The sputtering process was performed at 6 mTorr with 100 W RF power under Ar environment. After that, the conventional photolithography was carried out once more to align and expose the electrode areas. Next, for metallization, the Ti/Au (10 nm/200 nm) layers were deposited by using an e-beam evaporator (KVE-E2004, Korea Vacuum Tech., LTD), and then the lift-off process was followed. Meanwhile, the growth solution was made by dissolving the equimolar (5, 25, and 50 mM) zinc nitrate hexahydrate (Sigma Aldrich Co.) and hexamethylentetramine (Sigma Aldrich Co.) in DI water (200 mL) at room temperature. Herein, 1 mL of ethylenediamine (Sigma Aldrich Co.) was added into all the solutions to make the ZnO NRs with a high aspect ratio. The solutions were stirred with a magnetic bar for 2 h, which led to a homogeneous solution. After that, the as-prepared samples were immersed into the growth solution in an oven at 90 °C for 1.5 h. For passivation, the SiO_2_ was deposited on the as-fabricated devices with a deposition rate of ~0.15 nm/s. The morphology, elemental mappings, and crystallinity of the as-fabricated samples were observed by using a field-emission scanning electron microscope (FE-SEM) (LEO SUPRA 55, Carl Zeiss, Germany), an energy dispersive X-ray (EDX), and an X-ray diffractometer (XRD) (Mac Science, M18XHF-SRA), respectively. For the device characteristics of ZnO NR-based NUV PDs, the photocurrents were measured by using a dual channel source meter (Keithley 2636A) under the illumination (2.3 mW/cm^2^) using a NUV light-emitting diode operating at ~380 nm.

## Results and Discussion

**Fig. 2:**
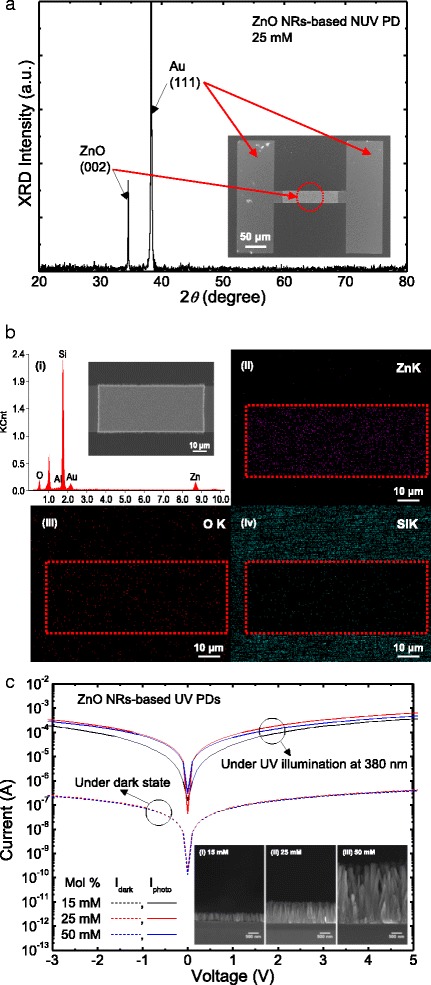
XRD, FE-SEM, EDX, and morphology-dependent *I-V* curves. **a** 2*θ* scan XRD pattern of the ZnO NRs grown at 25 mM in ZnO NR-based NUV PDs, **b** (*i*) EDX spectrum of the selectively grown ZnO NRs on the active channel and their corresponding (*ii*) Zn, (*iii*) O, and (*iv*) Si elemental mapping images, and **c** *I-V* curves of the ZnO NR-based NUV PDs at (*i*) 15 mM, (*ii*) 25 mM, and (*iii*) 50 mM under dark state and illumination of 380 nm light. The photograph of **a** shows the top-view FE-SEM image of the ZnO NR-based NUV PD. The inset of **c** shows the cross-sectional FE-SEM images of the ZnO NRs grown at different concentrations of 15, 25, and 50 mM

Figure 2a shows the 2*θ* scan XRD pattern of the ZnO NRs grown at 25 mM in ZnO NR-based NUV PDs. The ZnO XRD peak was observed at 2*θ* = 34.4° which corresponds to the (002) direction of hexagonal wurtzite ZnO from the JCPDS card no. 89-1397, and no other ZnO peaks were found. This indicates that the c-axis of hydrothermally grown ZnO NRs is oriented vertically to the AZO seed layer. The XRD peak observed at 2*θ* = 38.3° represented the (111) direction of Au electrodes. The photograph of Fig. 2a shows the top-view FE-SEM image of the ZnO NR-based NUV PD, grown at 25 mM, with the active channel width (*W*_ch_) of 90 μm. From the FE-SEM image, it was confirmed that the hydrothermally grown ZnO NRs were well aligned vertically between the Ti/Au electrodes.

Figure 2b shows the (i) EDX spectrum of the selectively grown ZnO NRs on the active channel and their corresponding (ii) zinc (Zn), (iii) oxygen (O), and (iv) silicon (Si) elemental mapping images. From the EDX spectrum, the amounts of zinc and oxygen atoms were quantified to be ~14.3 and 20.5 %, respectively, indicating a 1:1 ratio of Zn and O for ZnO NRs. The amount of oxygen atoms was a little higher than that of zinc atoms because the oxygen in SiO_2_ layer on Si substrate was also counted. The elemental mapping images exhibited that the zinc and oxygen atoms found at high concentrations were uniformly distributed over the active channel region while the silicon was rarely observed. This indicates that the ZnO NRs were densely grown on the active channel without any significant defects or vacancies.

Figure 2c shows the current-voltage (*I-V)* curves of the ZnO NR-based NUV PDs at (i) 15 mM, (ii) 25 mM, and (iii) 50 mM. Herein, the *W*_ch_ was fixed to be 30 μm. At the bias of 5 V, very low dark currents were observed from all the devices while the photocurrents were measured to be ~0.35, 0.63, and 0.46 mA for the solution concentrations of 15, 25, and 50 mM, respectively. It is well known that the magnitude of photocurrent is closely related to the surface area and crystallinity of ZnO NRs. The ZnO NRs grown at 25 mM provided a relatively large surface area. At 50 mM, on the other hand, the photocurrent decreased due to the reduced surface area and low crystallinity of large ZnO NRs [2, 16, 17]. The inset of Fig. 2c shows the corresponding cross-sectional FE-SEM images of the ZnO NRs grown at 15, 25, and 50 mM using the hydrothermal synthesis. The diameter and height of ZnO NRs were increased as the solution concentration was increased. These values were estimated to be 40 ± 5 nm (diameter)/385 ± 15 nm (height), 65 ± 10 nm/1000 ± 50 nm, and 205 ± 20 nm/2600 ± 300 nm, indicating the aspect ratio of 9.6, 15.4, and 12.7, for the solution concentrations of 15, 25, and 50 mM, respectively. This can be explained by the fact that the diffusion of Zn^2+^ ions to nuclei is more enhanced at higher solution concentration. At 50 mM, however, the aspect ratio was slightly decreased because the excessive Zn^2+^ ions cause the higher growth rate on the overall facets of ZnO, i.e., the rapid isotropic growth of ZnO NRs [2, 18, 19].

**Fig. 3:**
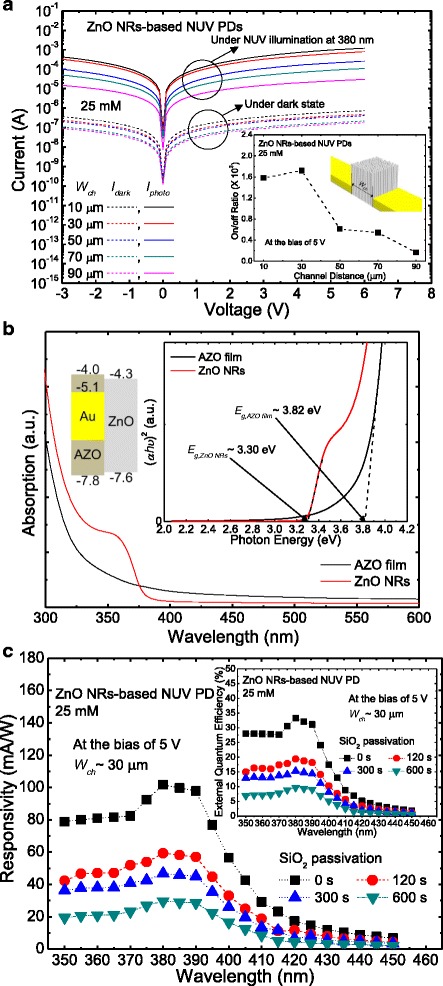
Active channel width-dependent device performance and spectral responsivity. **a** *I-V* curves of the ZnO NR-based NUV PDs grown at 25 mM with different *W*_ch_, **b** optical absorption spectra of the AZO thin films with/without ZnO NRs grown at 25 mM, and **c** spectral responsivity of the ZnO NR-based NUV PD grown at 25 mM with the *W*_ch_ of 30 μm. The inset of **a** shows the on/off ratio along the *W*_ch_. The inset of **b** shows the plot of (αhν)^2^ versus photon energy for the AZO thin films with/without ZnO NRs grown at 25 mM. The inset of **c** shows the EQE of the ZnO NR-based NUV PD grown at 25 mM with the *W*_ch_ of 30 μm

Figure 3a shows the *I-V* curves of the ZnO NR-based NUV PDs grown at 25 mM with different *W*_ch_ from 10 to 90 μm with an interval of 10 μm. At room temperature, under dark state, the electrons trapped in ZnO NRs can be excited to the conduction band by absorbing an ambient thermal energy and then driven to the electrodes by an externally applied potential (known as dark current). Under the illumination of light, the photocurrent is obtained from the electron and hole pairs generated by absorbing an incident light. As shown in Fig. 3a, both the dark current and photocurrent were reduced as the *W*_ch_ was increased. This behavior could be closely related to the carrier leakage and trapping into the AZO layer. The inset of Fig. 3a shows the on/off ratio of the ZnO NR-based NUV PDs grown at 25 mM as a function of *W*_ch_. The on/off ratio can be calculated by the well-known equation of *R* = (*I*_photo_ − *I*_dark_)/*I*_dark_. According to the behavior of dark current and photocurrent as mentioned above, it could be expected that the on/off ratio has a trade-off relationship with the *W*_ch_. At the bias of 5 V, as shown in inset of Fig. 3a, the on/off ratio was slightly increased until the *W*_ch_ became wider to 30 μm and then it decreased with further increase of the *W*_ch_, indicating ~1580, 1720, 612, 542, and 166 for the *W*_ch_ of 10, 30, 50, 70, and 90 μm, respectively, because the photocurrent was more reduced than the dark current. Above the *W*_ch_ = 30 μm, especially, the on/off ratio was significantly decreased in comparison with that at the *W*_ch_ = 10 μm. This may be attributed to the enhanced carrier leakage for longer *W*_ch_.

In order to explain the behavior of currents as mentioned above, the energy bandgap was determined from the optical absorption spectrum. Figure 3b shows the optical absorption spectra of the AZO thin films with/without ZnO NRs grown at 25 mM. The thickness of the deposited AZO thin film was ~50 nm. As shown in Fig. 3b, for both the samples, a strong absorption was observed in the wavelength region below 380 nm, which is well matched to their typical optical properties. In comparison with the ZnO NRs, the relatively low absorption was observed from the AZO thin film. The AZO thin film on the active channel would not much affect the photocurrent because it was gradually reduced during the growth of ZnO NRs as shown in the inset of Fig. 2c. The absorption spectrum of the AZO thin film was shown only to obtain its energy bandgap under the Ti/Au electrodes. The inset of Fig. 3b shows the plot of (αhν)^2^ versus photon energy for the AZO thin films with/without ZnO NRs grown at 25 mM. The energy bandgap is roughly evaluated by the following equation: [20, 21]

1 $$ {\left(\upalpha \mathrm{h}\upnu \right)}^2=A\left(\mathrm{h}\upnu -{E}_{\mathrm{gap}}\right) $$

where *α* is the absorption coefficient, hν is the photon energy, and *A* is the constant. The bandgap was extracted by extrapolating the linear line from the spectra, indicating ~3.30 and 3.82 eV for the ZnO NRs and AZO layer, respectively. The bandgap of ZnO NRs was estimated to be slightly lower than the typical ZnO bandgap of 3.37 eV while that of AZO layer to be higher. The bandgap of ZnO NRs could be reduced by the defects such as vacancies and interstitials [22, 23]. It is reported that the donor states below the conduction band can be formed by the divalent zinc vacancies, monovalent zinc interstitials, and neutral impurities while the acceptor states above the valance band by the monovalent vacancies of zinc and oxygen, leading to the narrowed bandgap [23]. The widen bandgap of AZO layer can be explained by the Burstein-Moss effect which represents that the increase of carrier concentration in degenerate semiconductors pushes the Fermi level up to higher energy states. For the AZO layer, its absorption edge was blue-shifted by the excess carriers generated from the Al dopants [24–27]. The work function of Au is typically reported to be 5.1–5.4 eV while that of sputtered AZO in Ar environment to be ~4.0–4.1 eV [28]. Thus, some of generated carriers could flow to the AZO layer through the relatively low potential barrier. As a result, the probability of current leakage could be increased as the active channel area was increased.

Figure 3c shows the spectral responsivity (*R*_*λ*_) of the unpassivated and passivated ZnO NR-based NUV PDs grown at 25 mM with the *W*_ch_ of 30 μm as a function of wavelength of incident light. The SiO_2_ passivation was performed at different deposition times of 120, 300, and 600 s to reduce the surface defects of ZnO NRs and environmental effects. The *R*_*λ*_ indicates the ratio of photocurrent to light intensity at certain wavelength. For measurements, the monochromatic light source was used from 350 to 450 nm with an interval of 5 nm. At the bias of 5 V, for all devices, the *R*_*λ*_ exhibited relatively high values below 390 nm while it was gradually decreased above 400 nm, which exhibits similar tendency with typical absorption spectra of ZnO NRs. Though the passivation on ZnO NRs with dielectric or organic materials enables enhanced photoconduction and efficiency by reducing the probability of surface recombination [29–31], for the ZnO NR-based NUV PDs, the *R*_*λ*_ was dramatically dropped down with increasing the SiO_2_ deposition time for passivation. This is mainly attributed to the high resistivity of SiO_2_ [31]. The optical losses by SiO_2_ passivation are not considered because it is highly transparent (i.e., transmittance > 90 %) in the NUV and visible ranges. At the wavelength of 380 nm, the highest *R*_*λ*_ values were obtained to be 101.68, 59.29, 46.80, and 29.62 mA/W for the devices passivated with SiO_2_ deposited at the deposition times of 0, 120, 300, and 600 s, respectively. The inset of Fig. 3c shows the external quantum efficiency (EQE) of the unpassivated and passivated ZnO NR-based NUV PDs grown at 25 mM with the *W*_ch_ of 30 μm as a function of wavelength of incident light. The EQE of ZnO NR-based NUV PDs was calculated using the equation of EQE = 1240 × *R*_*λ*_ × *λ*^− 1^. At 380 nm, as shown in the inset of Fig. 3c, the maximum EQE values were observed to be 33.2, 19.4, 15.3, and 9.7 % for the devices passivated with SiO_2_ at 0, 120, 300, and 600 s, respectively.

**Fig. 4:**
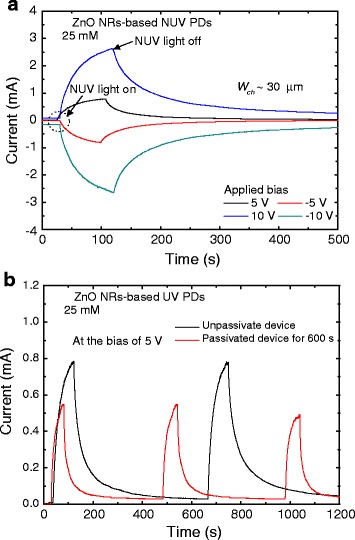
Photoresponse characteristics and SiO_2_ passivation effect. **a** Photoresponse characteristics of the ZnO NR-based NUV PD grown at 25 mM with the *W*_ch_ of 30 μm at different biases and **b** SiO_2_ passivation effect on the photoresponse property of the ZnO NR-based NUV PD grown at 25 mM with the *W*_ch_ of 30 μm

Figure 4a shows the photoresponse characteristics of the ZnO NR-based NUV PD grown at 25 mM with the *W*_ch_ of 30 μm at different biases. The decay time of photo-generated carriers is closely related to the reabsorption of oxygen and water molecules at the surface of ZnO NRs. Typically, natural ZnO has an *n*-type conductivity. This may be attributed to the shallow donors with substitutional hydrogens at oxygen vacancies [32–34]. The interstitial hydrogens have been discussed to explain the unintentional conductivity of ZnO [35, 36]. Under the dark state, the oxygen and water molecules in the ambient air are combined with pre-existing free electrons of ZnO NRs. Thus, O_2_ molecules become O^2−^ ions at the surface of ZnO NRs, leading to the reduced carrier density and mobility in ZnO NRs. Under the UV illumination, the carrier density is increased by the photo-generated electron and hole pairs. Meanwhile, some of holes move to the surface and recombine with the O^2−^ ions, which results in the release of O_2_ molecules. For this reason, slow photoresponse properties (i.e., decay time) can be observed in ZnO NR-based UV PDs [36–38]. As shown in Fig. 4a, the symmetric trend at positive and negative biases is due to the MSM structure. The photoresponse properties can be characterized using the rise and reset times. The rise time indicates the required time to reach 90 % of maximum photocurrent while the reset time for the required time to recover 1/*e* times (37 %) of the maximum photocurrent. At the bias of 5 V, the rise and reset times were evaluated to be 55.5 and 33.1 s, respectively.

Figure 4b shows the SiO_2_ passivation effect on the photoresponse property of the ZnO NR-based NUV PD grown at 25 mM with the *W*_ch_ of 30 μm. The SiO_2_ passivation was performed at the deposition time of 600 s. The SiO_2_ passivation was carried out to prevent the absorption and reabsorption of oxygen and water molecules to ZnO NRs. As shown in Fig. 4b, the decay time of the passivated device became shorter than that of the unpassivated device. For the passivated device, at the bias of 5 V, the rise and reset times were decreased down to be 33.8 and 13.9 s, respectively. Compared to the unpassivated device, it could be found that the photocurrent of the passivated device was less generated because the SiO_2_ passivation could increase the total resistivity of the device. Herein, the comparison between this and other ZnO-based UV PDs is summarized in Table 1.

**Table 1:** Comparison of device properties between this and other ZnO-based UV PDs

Materials	Wavelength (nm)	Responsivity (mA/W)	Rise/fall time (s)	Bias voltage (V)	Reference
ZnO NRs-phenanthrene	365	2.0 × 10^7^	–	2	[7]
ZnO NPs-graphene core-shell	375	640 × 10^3^	0.009/0.011	20	[39]
ZnO NRs-PVK-Cu_2_O	360	13.28 × 10^3^	8.7/128.3	−0.5	[8]
ZnO NRs between asymmetry Au	370	20	–	0	[3]
ZnO NCs	350	>8.5	0.5/1	0	[6]
ZnO NRs on AZO	380	102	55.5/33.1	5	This work

## Conclusions

The ZnO NR-based NUV PDs in the type of MSM structure were successfully fabricated by the conventional photolithography and hydrothermal synthesis processes. The device characteristics were analyzed and optimized with various solution concentrations for different *W*_ch_. The optimized device performance was achieved from the ZnO NRs grown at 25 mM with the *W*_ch_ of 30 μm, indicating the photocurrent of 0.63 mA and on/off ratio of 1720. The SiO_2_ passivation enhanced the photoresponse properties (i.e., reduced rise and reset times), but it made the photocurrent reduced. These results may be helpful for the facile fabrication and optimization of device performance in ZnO NR-based UV PD applications.
